# Supplementary material for: New Insight into the Concanavalin A-Induced Apoptosis in Hepatocyte of an Animal Model: Possible Involvement of Caspase-Independent Pathway
Source: Molecules. 2023 Jan 30;28(3):1312. doi: 10.3390/molecules28031312 (PMC9919242; doi:10.3390/molecules28031312)
Supplement: Supplementary file 1 [file molecules-28-01312-s001.zip › molecules-2119199-supplementary.pdf]

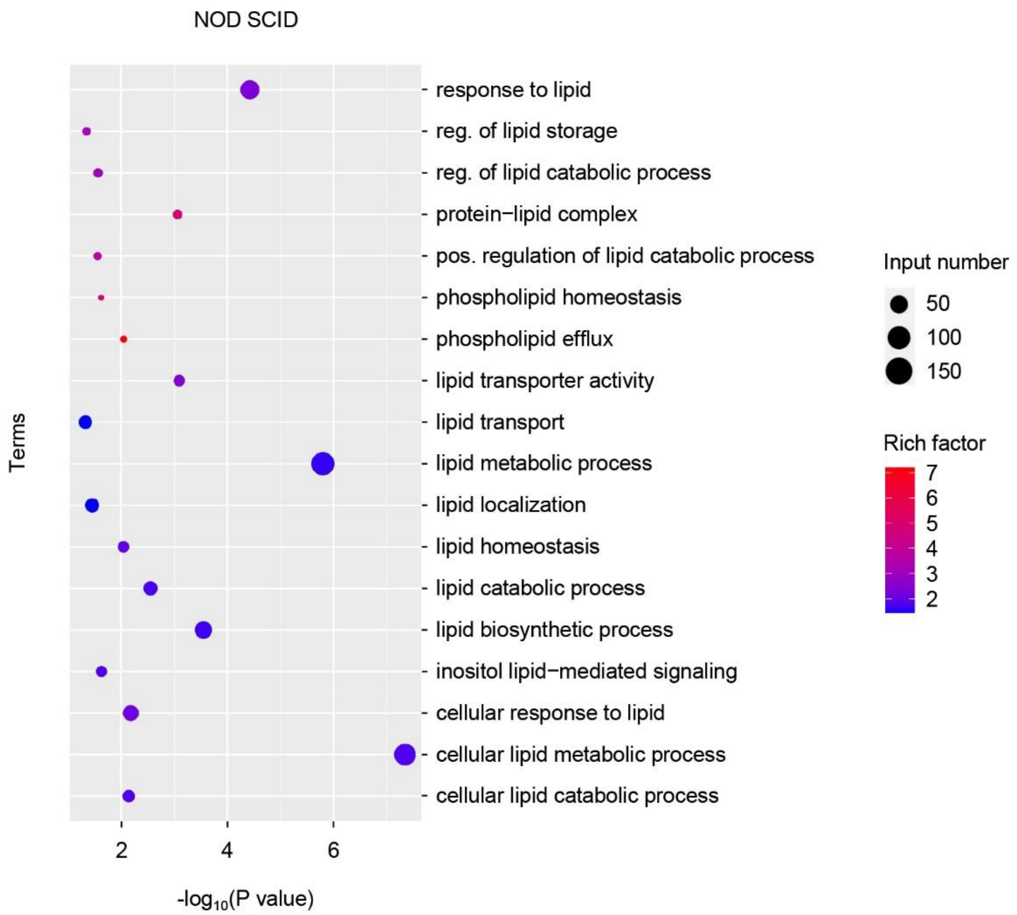

Figure S1. Down-regulation of lipid metabolism-related pathways is enriched in the liver of Con A-injected NOD SCID mice.

RNA was obtained from the livers of NOD SCID mice 8 h after Con A (25 mg/kg) or vehicle ad-ministration, then analyzed by RNA-Seq or characterization. GO analysis of DEGs shows the up- and down-regulated pathways related to lipid response and metabolism.
